# Overexpression of Krüppel-Like Factor 9 Enhances the Antitumor Properties of Paclitaxel in Malignant Melanoma-Derived Cell Lines

**DOI:** 10.3390/ph16040557

**Published:** 2023-04-06

**Authors:** Mohammed O. Altonsy, George X. Song-Zhao, Mahmoud M. Mostafa, Paule Régine Mydlarski

**Affiliations:** 1 Division of Dermatology, Department of Medicine, University of Calgary, Calgary, AB T2T 5C7, Canada; george.songzhao@ucalgary.ca; 2 Snyder Institute for Chronic Diseases, University of Calgary, Calgary, AB T2N 4N1, Canada; mahmoud.mostafa@ucalgary.ca; 3 Department of Zoology, Faculty of Science, Sohag University, Sohag 82524, Egypt

**Keywords:** melanoma, chemotherapy, paclitaxel, KLF-9, combination-therapy

## Abstract

Over the past decade, the treatment of metastatic melanoma has improved significantly due to the development of innovative therapies, such as drugs that target the BRAF/MAPK kinase pathway and the PD-1 pathway. However, these therapies do not work for all patients, highlighting the need for additional research on the pathophysiology of melanoma. Paclitaxel is a chemotherapeutic agent used when first-line treatments are unsuccessful; however, its efficacy is limited. Since Krüppel-like factor 9 (KLF9) (antioxidant repressor) is downregulated in melanoma, we propose that restoring KLF9 levels may sensitize malignant melanoma to chemotherapeutic agents, such as paclitaxel. We used adenovirus overexpression and siRNA technologies to assess the role of KLF9 in mediating the response of malignant melanoma-derived cell lines RPMI-7951 and A375 to paclitaxel treatment. We found that increasing KLF9 levels potentiates the effectiveness of paclitaxel, as shown by apoptotic parameters such as decreased cell viability, pro-caspase-3 activation, increased number of annexin V-positive cells, and reduction in nuclear proliferation marker (KI67). These results suggest that KLF9 may be a potential target for improving chemotherapeutic response in melanoma.

## 1. Introduction

Melanoma develops from melanin-producing cells in the skin, mucous membranes, nails, and eyes. Genetic and environmental factors, such as DNA damage resulting from exposure to ultraviolet light, contribute to the progression of melanoma. In fact, melanoma has one of the highest mutation rates of any cancer type, which contributes to its diversity and resistance to treatment. Previous research has led to the identification of several pathways, such as the BRAF/MAPK kinase and the PD-1 pathways, resulting in novel immunotherapies like nivolumab and ipilimumab. These therapeutic advancements have resulted in the transformation of metastatic melanoma from a disease with a dismal five-year survival into one with a near 50% survival rate [1]. Taken together, this data highlights the importance of better understanding the molecular mechanisms of malignant melanoma that could lead to revolutionary treatment strategies.

Krüppel-like factor 9 (KLF9) is a transcription factor ubiquitously expressed in human skin, brain, lung, kidney, and intestines [2,3,4,5,6]. In vitro and in vivo studies using human cells and animal models have demonstrated that KLF9 regulates the proliferation of keratinocytes, B-cells, and neurons [7,8,9]. Interestingly, KLF9 downregulation is seen in various human cancers, including melanoma, and high KLF9 mRNA levels correlate with increased survival rates in melanoma patients [10,11,12,13]. Furthermore, KLF9 represses many antioxidant agents that regulate reactive oxygen species (ROS), which are essential for suppressing malignant transformation and promoting apoptosis in transformed cells [12,14]. KLF9-dependent ROS repression may be crucial for cancer progression as ROS levels are higher in cancer due to increased metabolic activity [15].

Cancer cells, in contrast to normal cells, evade the intracellular and intercellular signals that control normal cell division, resulting in continuous and uncontrolled cell growth. Chemotherapeutic agents target dividing malignant cells and disrupt their multiplication machinery, ultimately leading to cancer cell death [16,17,18]. Paclitaxel is a chemotherapeutic agent known to promote and stabilize the assembly of microtubules and halt the process of microtubule disassembly, leading to cell growth arrest [19]. It has been successfully used for treating different types of cancers since 1989 [20]. Unfortunately, paclitaxel treatment of malignant melanoma only results in minor improvement or resistance [21,22]. The combination therapy using paclitaxel and carboplatin is the currently recommended option for patients who are not candidates for immunotherapy [23], such as nivolumab and ipilimumab, or targeted therapy, such as monoclonal antibodies and tyrosine kinase inhibitors.

Herein, we hypothesize that the KLF9-downregulation, seen in melanoma patients, may contribute to paclitaxel resistance. We therefore used KLF9 overexpression and knockdown systems to determine the mechanism by which KLF9 affects paclitaxel-induced apoptosis and cell replication inhibition in melanoma cell lines.

## 2. Results

### 2.1. A Rapid Proliferation of Malignant Melanoma-Derived Cell Lines Compared to Normal Melanocytes

To determine the cell number corresponding to a given MTT absorbance value, we prepared serial dilutions of primary melanocytes, RPMI-7951, and A375 cells and grew them for 24 h before conducting the MTT assay. Cell numbers and absorbance values were plotted to extract a second-order polynomial equation (Figure S1A), and to extrapolate cell numbers [*C*] from a given absorbance value (*A*). Subsequently, to compare the growth rates between primary and malignant cell types, we plated a fixed number of 30,000 cells in each well of 48-well plates, and MTT assays were carried out at 24, 48, 72 and 96 h (Figure S1B). We observed that the number of primary melanocytes remained almost unchanged over the first three days and slightly but significantly increased by 16.41% (*p* ≤ 0.01) on day four. In contrast, RPMI-7951 and A375 cells exhibited a rapid growth rate, as cell numbers almost doubled after 72 h (Figure S1B). These data confirmed that the cell models we used were suitable to study the effect of paclitaxel treatment on normal versus rapidly dividing skin cells.

### 2.2. A Dose- and Time-Dependent Effect of Paclitaxel on Cellular Viability

**Figure 1:**
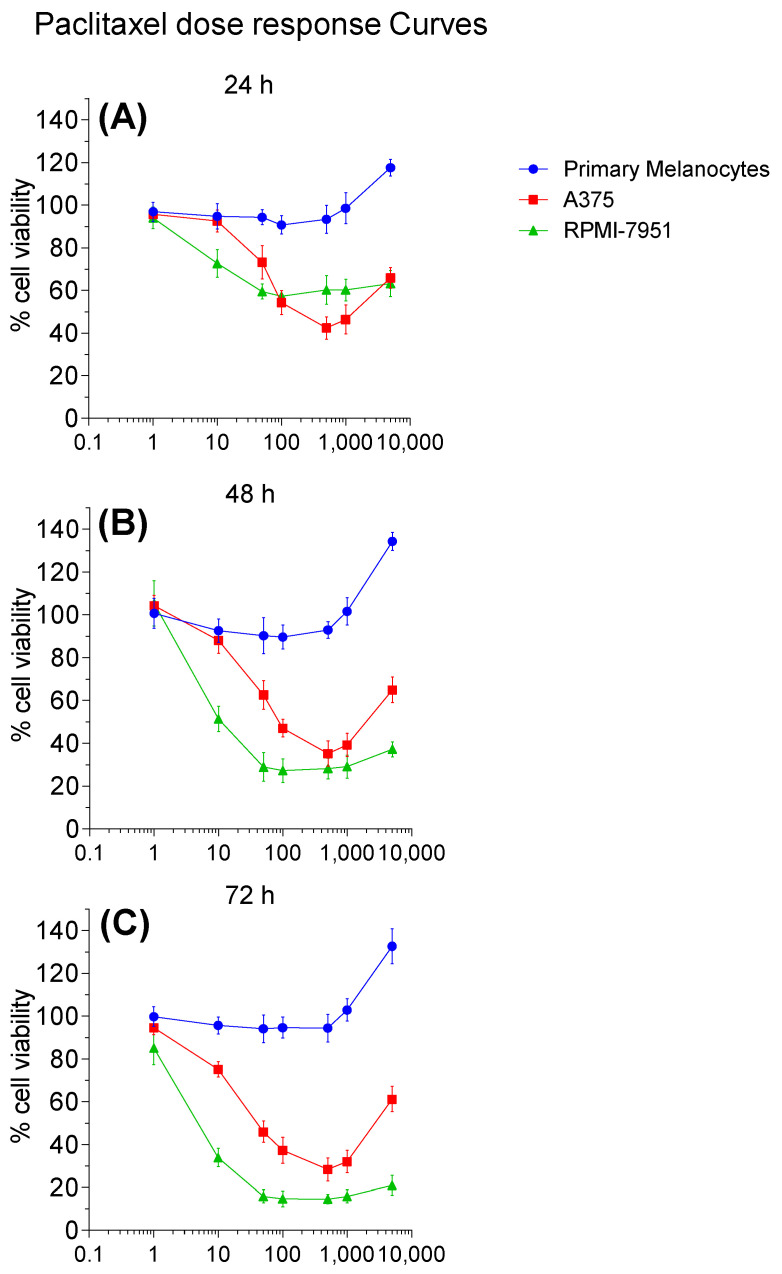
Cell viability responses to paclitaxel treatment in primary melanocytes and malignant melanoma-derived cell lines. The MTT assay illustrates dose–response curves of the primary melanocytes, RPMI-7951 and A375 cells to different concentrations of paclitaxel, ranging from 1 nm to 5 µM. The effect of paclitaxel on cellular growth was assessed at 24 (**A**), 48 (**B**), and 72 h (**C**) following the treatment. Absorbance was measured at 590 nM, and data (*n* = 5–6) was expressed as mean OD values with SD, using Graph-Pad Prism software.

To study the effect of paclitaxel on cell viability of primary and malignant melanoma cells, we used varying concentrations (1, 10, 50, 100, 500, 1000, and 5000 nM) of paclitaxel to treat the cells. Subsequently, we quantified the cell viability using the MTT assay. The absorbance values of MTT were measured, and cell numbers were determined every 24 h for three consecutive days (Figure 1, and IC_50_ data Supplementary Table S1). Paclitaxel did not affect the viability of primary melanocytes treated with concentrations up to 0.5 µM; interestingly, increasing paclitaxel concentration to 1 and 5 µM corresponded to increased absorbance values in primary melanocytes, regardless of the treatment time. In contrast to primary melanocytes, malignant melanoma cells, RPMI-7951 and A375 demonstrated a dose-and time-dependent decrease in viability. The effect was more pronounced in RPMI-7951 cells at concentrations lower than 100 nM, compared to A375 cells. In RPMI-7951, the percentages of viable cells did not change significantly in treatments using concentrations higher than 50 nM, demonstrating a plateau effect in cell viability curves (Figure 1). In A375 cells, the decline in cell viability continued with increasing paclitaxel concentrations up to 500 nM. Similar to primary melanocytes, A375 cells showed reverse effects in response to higher doses (1, and 5 µM) of paclitaxel, where the absorbance curves trended upwards again at all time points.

### 2.3. Paclitaxel Increased KLF9 mRNA and Protein Expression in RPMI-7951 and A375 Cells

**Figure 2:**
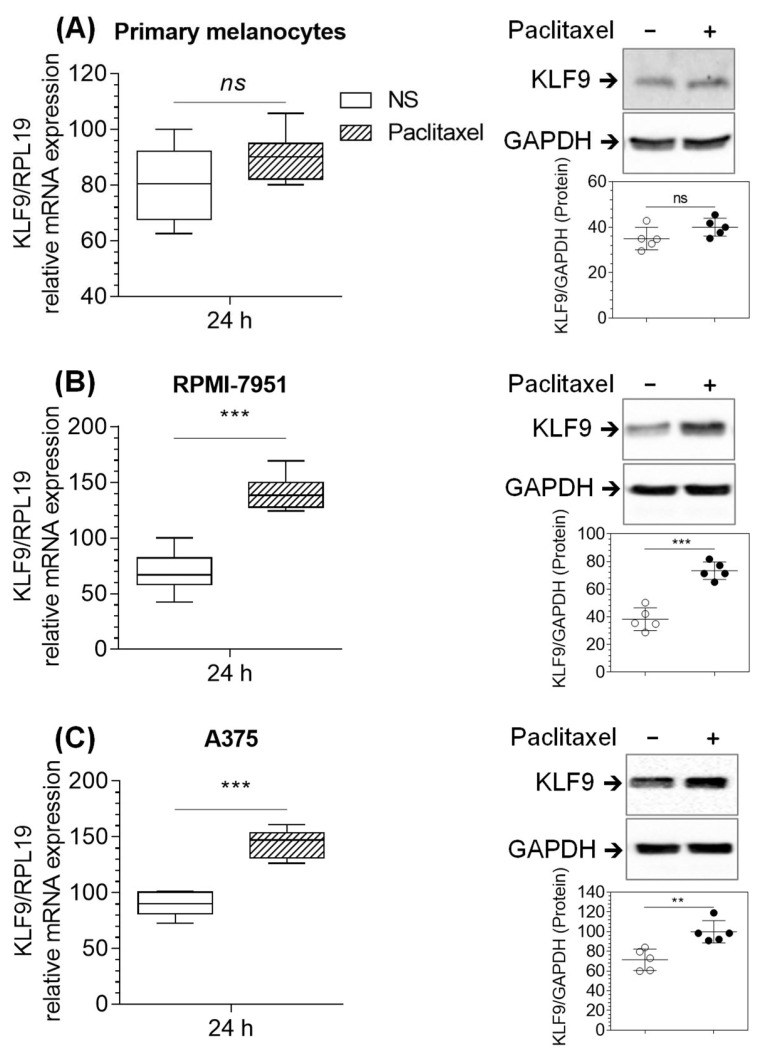
Paclitaxel increased the expression of KLF9 (mRNA and protein) in RPMI-7951 and A375 cells but not in primary melanocytes. RT-PCR (left panels) and Western blot analysis (right panels) of KLF9 mRNA and protein, respectively. (**A**) primary melanocytes, (**B**) RPMI-7951, and (**C**) A375 cells. For KLF9 mRNA and protein analysis, cells were treated with paclitaxel (100 nM) and harvested after 24 h for RT-PCR or Western blot, as described in the methods section. Relative quantities of KLF9-mRNA were normalized to mRNA quantities of the housekeeping gene, RPL19, within the same sample. For protein analysis, cell lysates (50 µg total protein) were electrophoresed, transblotted, and immunoprobed against KLF9 or GAPDH (loading control) antibodies. Densitometric analysis of protein bands (right bottom scatter plots) was calculated as mean differences of the densitometric arbitrary scan units of KLF9 bands normalized to GAPDH. Data (*n* = 5–6), box and whiskers, left panels, and scatter plots, right panels, represent means (±SD). ^ns^ *p* > 0.05, ** *p* ≤ 0.01, *** *p* ≤ 0.001 vs. none-stimulated cells.

To explore whether paclitaxel regulates KLF9 expression, we treated primary melanocytes and malignant melanoma cell lines with 100 nM paclitaxel for 24 h. RT-PCR data demonstrated that KLF9 mRNA levels remained unchanged in primary melanocytes, whereas the levels increased by 102.8% (*p* ≤ 0.001), and 46.5% (*p* ≤ 0.001) in RPMI-7951 and A375 cells, respectively, as compared to non-stimulated cells (Figure 2A–C left panels). KLF9 protein followed similar patterns to the mRNA, showing an increase of 104.3% (*p* ≤ 0.001), and 34.6% (*p* ≤ 0.01) in RPMI-7951 and A375 cells, respectively, in comparison to non-stimulated cells (Figure 2A–C right panels). Thus, paclitaxel regulation of KLF9 is selective to malignant melanoma cells.

### 2.4. KLF9 Modulated Paclitaxel-Induced Apoptosis in RPMI-7951 and A375 Cells

**Figure 3:**
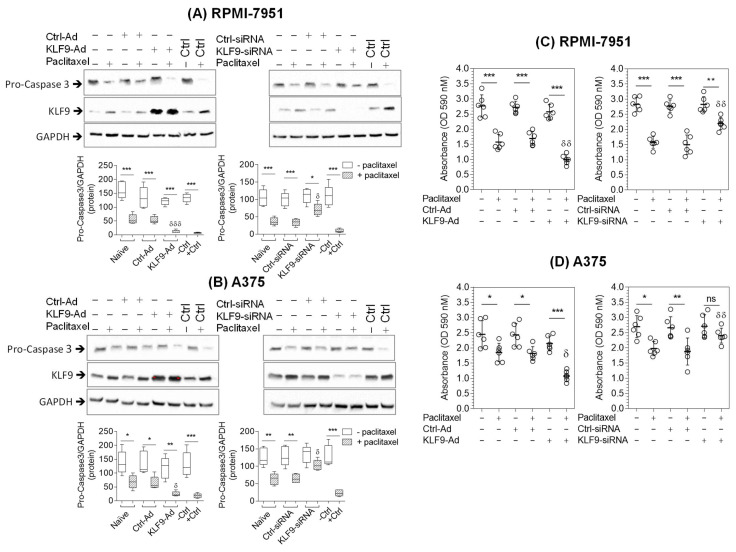
KLF9 overexpression augmented the paclitaxel apoptotic effect, while KLF9 knockdown attenuated such an effect on RPMI-7951 and A375 cells. Western blot analysis of cell lysates prepared from RPMI-7951 (**A**) or A375 cells (**B**). Cells were transduced with KLF9-overexpressing adenovirus (KLF9-Ad) or transfected with KLF9-siRNA, followed by 24 h treatment with paclitaxel (100 nM). Negative (-Ctrl) or positive control (+Ctrl) samples were prepared from naïve untreated or paclitaxel treated cells (100 nM for 72 h), respectively. Box and whiskers graphs (left-bottom) illustrate the densitometric analysis of caspase-3 band intensity. The MTT assay was performed as described in the materials and method section. The MTT assay shows the effects of adenoviral overexpression or siRNA knockdown of KLF9 on cell viability in RPMI-7951 (**C**) or A375 cells (**D**) treated with paclitaxel (100 nM) for 24 h before reading the MTT absorbance. Data (*n* = 6), scatter plots, right panels, represent means (±SD). ^ns^ *p* > 0.05, * *p* ≤ 0.05, ** *p* ≤ 0.01, *** *p* ≤ 0.001. ^δ^ *p* ≤ 0.05, ^δδ^ *p* ≤ 0.01, ^δδδ^ *p* ≤ 0.001 vs. the absorbance values of naïve cells treated with paclitaxel.

Our data suggested that paclitaxel selectively reduced cell viability and upregulated KLF9 expression in malignant melanoma cells. We, therefore, investigated whether KLF9 plays a role in the paclitaxel-induced reduction in cell viability and apoptosis in RPMI-7951 and A375 cells. To study the role of KLF9, we compared paclitaxel effects in two different systems, where we overexpressed KLF9 protein in one and knocked it down in the other using KLF9-adenovirus and KLF9-siRNA, respectively. Western blot analysis was employed to measure caspase-3 activation and MTT assay to measure cell viability. Paclitaxel treatment significantly reduced pro-caspase-3 protein in RPMI-7951 cells by 65.17% (*p* ≤ 0.004) in naive cells, and by 59.87% (*p* ≤ 0.009) in control-adenovirus-transduced (ctrl-ad) cells when compared to non-stimulated cells. The effect of paclitaxel was more potent in KLF9-overexpressing cells, which showed a reduction of pro-caspase-3 protein by 90.9% (*p* ≤ 0.0002). This reduction was significantly lower than the reduction seen in paclitaxel-treated naive cells by 28.3% (*p* ≤ 0.001) and ctrl-ad cells by 34.13% (*p* ≤ 0.005). In contrast, paclitaxel activation of pro-caspase-3 was significantly reduced in KLF9-knockdown cells by 38.13% (*p* ≤ 0.02), when compared to naive cells by 64.97% (*p* ≤ 0.0007) and control siRNA-transfected (ctrl-siRNA) cells by 66.19% (*p* ≤ 0.0006) (Figure 3A).

Like RPMI-7951 cells, A375 cells also showed a similar reduction in pro-caspase-3 levels after paclitaxel treatment, with 64.46% (*p* ≤ 0.003) reduction in naive cells and 66.43% (*p* ≤ 0.01) reduction in ctrl-ad cells, respectively. The reduction in pro-caspase-3 levels increased significantly to 89.52% (*p* ≤ 0.002) in KLF9-overexpressing cells. Furthermore, A375 cells transfected with KLF9-siRNA showed diminished paclitaxel effects on the activation of pro-caspase-3, and there was no significant difference in pro-caspase-3 levels before and after paclitaxel treatment (*p* ≥ 0.05). However, paclitaxel reduced pro-caspase-3 protein by 54.82% (*p* ≤ 0.0007) in naive cells and by 54.43% (*p* ≤ 0.003) in ctrl-siRNA cells, respectively (Figure 3B). These data indicate that KLF9 overexpression augmented paclitaxel-induced activation of pro-caspase-3, whereas KLF9-knockdown attenuated or completely diminished paclitaxel apoptotic effects in RPMI-7951 or A375 malignant melanoma cell lines, respectively.

The role of KLF9 on paclitaxel-induced reduction in cell viability in malignant melanoma cell lines was further assayed using the MTT test (Figure 3C). In this experiment, we observed that paclitaxel significantly reduced RPMI-7951 cell viability in naïve and ctrl-ad cells to 56.92% (*p* ≤ 0.0008) and 62.12% (*p* ≤ 0.0006), respectively, when compared to non-stimulated cells. These effects were more pronounced in the KLF9-overexpression condition, where the number of viable cells decreased to 38.69% (*p* ≤ 0.0002) compared to untreated cells. Meanwhile, KLF9-knockdown attenuated paclitaxel effects, resulting in a decrease in cell viability to 77.63% (*p* ≤ 0.001), which was significantly higher than that seen in naïve, or ctrl-siRNA cells treated with the same concentration of paclitaxel for the same duration. In A375 cells (Figure 3D), paclitaxel significantly reduced viable cells to 75.61% (*p* ≤ 0.01) and 74.66% (*p* ≤ 0.01) in naïve and ctrl-ad cells, respectively. Whereas KLF9-overexpression augmented paclitaxel effects and reduced viable A375 cells to 50.19% (*p* ≤ 0.0001) compared to non-treated cells. We also explored the effect of KLF9-siRNA on paclitaxel outcomes in A375 cells and observed that paclitaxel did not significantly reduce (*p* ≥ 0.05) cell viability in KLF9-knockdown cells; in contrast, it significantly reduced cell viability in naïve, 73.23% (*p* ≤ 0.009), and ctrl-siRNA transfected cells, 70.59% (*p* ≤ 0.02) in the same experiment.

**Figure 4:**
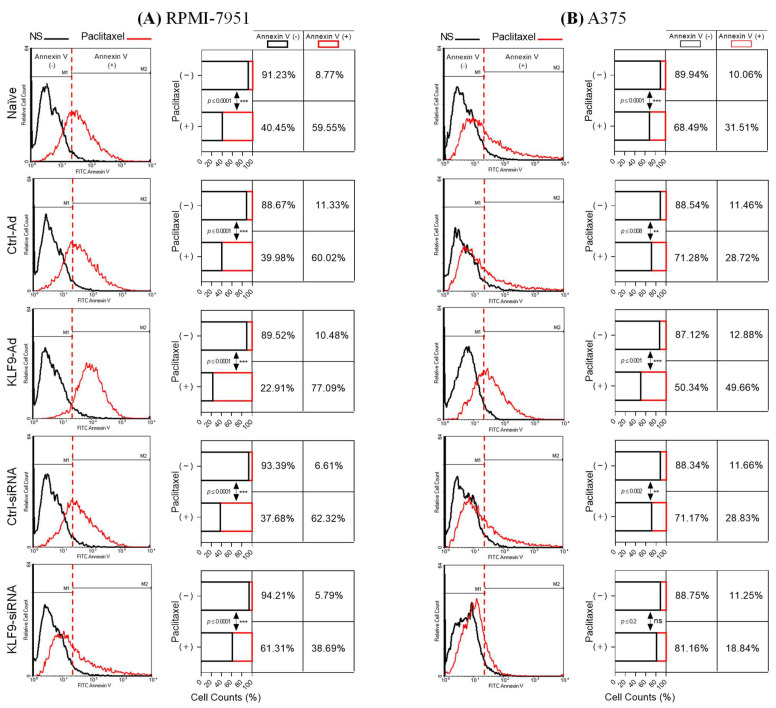
Flowcytometry analysis of malignant melanoma cells stained with annexin V-FITC apoptosis detection dye. Flow cytometry histograms (**left** panels) and stacked bar graphs (**right** panels) depict the effect of KLF9 adenoviral overexpression or siRNA knockdown on paclitaxel-induced apoptosis in RPMI-7951 (**A**) or A375 cells (**B**). Twenty-four hours post-viral transduction or siRNA transfection, cells were treated with paclitaxel (100 nM, for 24 h). Annexin V-FITC staining was performed as described in the methods section. Data (*n* = 5), ^ns^ *p* > 0.05, ** *p* ≤ 0.01, *** *p* ≤ 0.001 vs. none-stimulated cells.

KLF9 modulation of paclitaxel-induced apoptosis was also quantified with flow cytometry analysis of apoptotic cells using annexin V dye (Figure 4A,B). KLF9-overexpression significantly increased annexin V (+) cells to 77.09% (*p* ≤ 0.0001), and 49.66% (*p* ≤ 0.001) in RPMI-7951 and A375 cells, respectively. These numbers were significantly higher than naïve cells treated with paclitaxel by 17.53% (*p* ≤ 0.001) and 18.14%, (*p* ≤ 0.003) in RPMI-7951 and A375 cells, respectively. However, in KLF9-siRNA knockdown cells, annexin V (+) cells decreased to 38.68% (*p* ≤ 0.001) in RPMI-7951, and there was no significant difference between annexin V (+) cell numbers in non-stimulated and paclitaxel-treated A375 cells (*p* ≤ 0.2) as compared to cells transfected with ctrl-siRNA, which showed similar paclitaxel effects to naïve cells (Figure 4B).

### 2.5. KLF9 Modulated Paclitaxel Anti-Proliferative Effect in RPMI-7951 and A375 Cells

**Figure 5:**
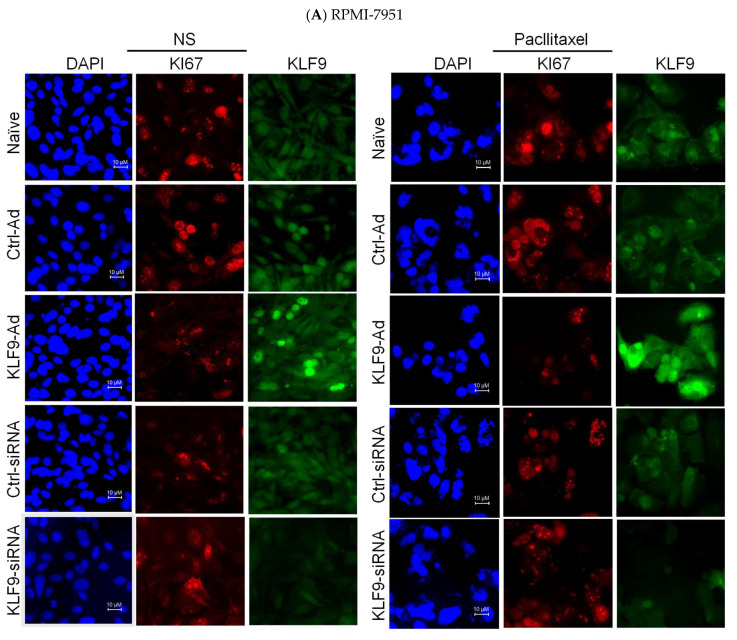

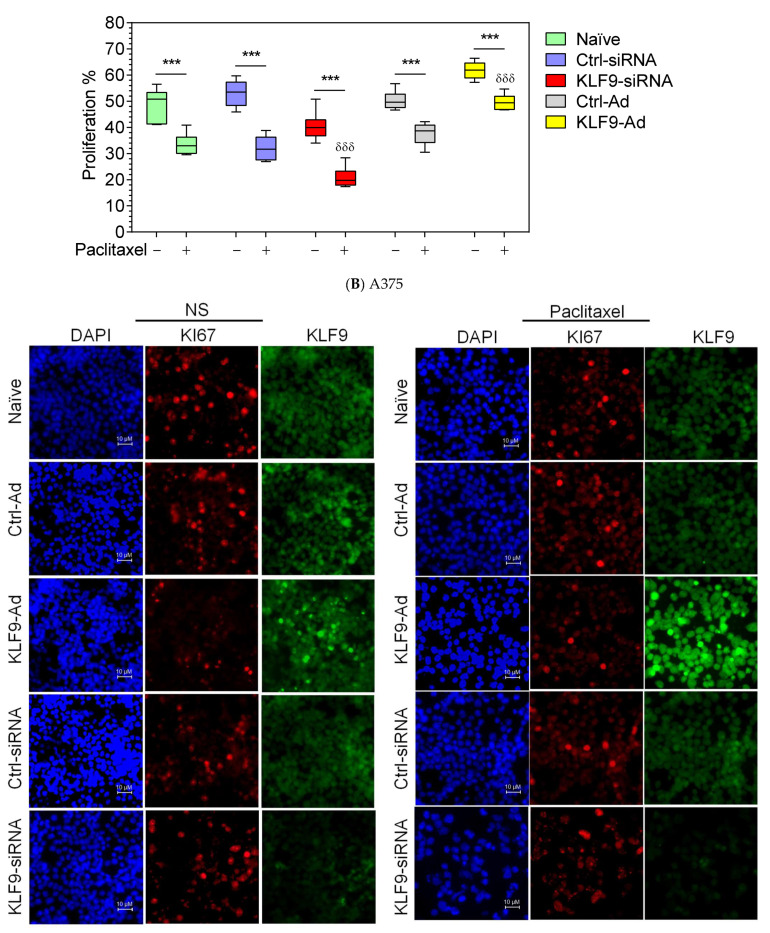

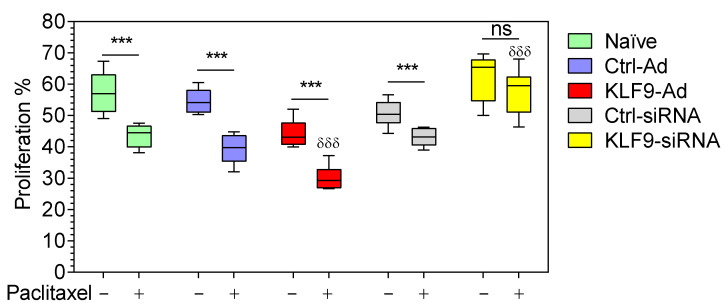
Fluorescent micrographs illustrate how KLF9 modulates paclitaxel’s effect on proliferation in malignant melanoma-derived cell lines. RPMI-7951 ((**A**) top graphs) and A375 cells ((**B**) top graphs) were plated on cover slips 24 h before viral transduction or siRNA transfection. Then, the cell monolayers were incubated for another 24 h followed by paclitaxel treatment (100 nM for 24 h). Cells were immunoprobed against KI67 (red) and KLF9 (green) antibodies, DAPI (blue) was used as a nuclear marker. Images were captured using an OLYMPUS IX73 inverted fluorescent microscope and cellSens Dimension 1.9 software (original magnification: 600; scale bar = 10 µM). KI67 fluorescence quantification was carried out using *imagej* online software, as described in the methods section. The extracted values for KI67 positive nuclei were plotted using Graph-Pad Prism software (box and whiskers bottom graphs). Significant difference was determined using ANOVA with Tukey’s correction for multiple comparisons. ^ns^ *p* > 0.05, *** *p* ≤ 0.001 vs. non-stimulated cells, ^δδδ^ *p* ≤ 0.001 vs. paclitaxel-treated naïve cells.

To investigate the effect of KLF9 overexpression or knockdown on cell proliferation and to study the response of paclitaxel’s anti-proliferative effects in malignant melanoma cells, we stained RPMI-7951 and A375 cells with the nuclear proliferation marker (Ki67). In RPMI-7951 cells, the quantification of Ki67 positive cells revealed that paclitaxel reduced cell proliferation to 68.84% (*p* ≤ 0.001), and 60.43% (*p* ≤ 0.001) in naïve and ctrl-ad cells; and as expected, those numbers were reduced to 51.56% (*p* ≤ 0.002) in KLF9-ad cells. Such an effect was attenuated to 80.38% (*p* ≤ 0.008), in KLF9-siRNA cells, but not in the ctrl-siRNA transfected cells after paclitaxel treatment. Moreover, in non-stimulated cells, the numbers of Ki67 positive nuclei were increased by 12.68% (*p* ≤ 0.01), in KLF9-siRNA knockdown cells compared to naïve cells (Figure 5A, and Supplementary Table S2). In A375 cells, paclitaxel reduced proliferation of naïve and ctrl-ad cells to 76.03% (*p* ≤ 0.003), and 72% (*p* ≤ 0.006), respectively. Again, in KLF9-overexpressing cells, proliferation was significantly reduced to 68.16% (*p* ≤ 0.001). This level of reduction is significantly less than in naïve cells, which received the same paclitaxel treatment by 10.35% (*p* ≤ 0.001). Unlike in RPMI-7951 cells, where the KLF9 knockdown attenuated the paclitaxel effect, in A375 cells, the knockdown diminished such an effect, as there was no significant difference in proliferation between non-stimulated and paclitaxel-treated cells. (Figure 5B, and Supplementary Table S3).

### 2.6. KLF9 Overexpression Potentiated Temozolomide, Cisplatin, and Carboplatin Apoptotic Effect on Malignant Melanoma Cells

**Figure 6:**
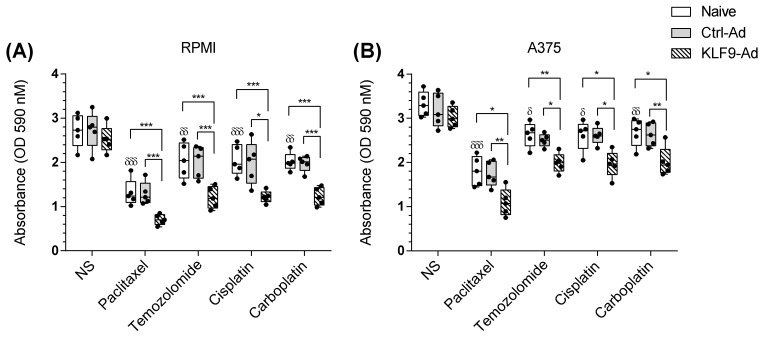
KLF9 overexpression potentiated TMZ, CIS, or CARB effects on cell viability in malignant melanoma-derived cell lines. Naïve, control-adenovirus (ctrl-ad) or KLF9-adenovirus transduced (KLF9-ad) RPMI-7951 (**A**) and A375 cells (**B**) treated with paclitaxel, TMZ, CIS, or CARB, 100 nM for 24 h, showed differential effects on cell viability. Data (*n* = 5), significant difference was determined using ANOVA with Tukey’s correction for multiple comparisons. * *p* ≤ 0.05, ** *p* ≤ 0.01, *** *p* ≤ 0.001 vs. non-stimulated cells, ^δ^ *p* ≤ 0.05, ^δδ^ *p* ≤ 0.01, ^δδδ^ *p* ≤ 0.001 vs. naïve non-stimulated cells.

The data clearly indicate that KLF9-overexpression significantly enhanced paclitaxel anti-cancer properties. Thus, we predicted that similar effects might apply to other chemotherapeutic agents widely used in malignant melanoma. We therefore treated RPMI-7951 and A375, naïve, ctrl-ad, or KLF9-ad cells, with temozolomide (TMZ), cisplatin (CIS), or carboplatin (CARB), (100 nM) for 24 h and performed cell viability assays. Paclitaxel was used as a positive control in this experiment (Figure 6A,B). Our results show that TMZ, CIS, or CARB reduced cell viability to 75.0%, (*p* ≤ 0.001), 75.52%, (*p* ≤ 0.005), 74.03, (*p* ≤ 0.001), in naïve RPMI-7951 cells. Similar results were obtained in ctrl-ad cells, treated with TMZ 73.97% (*p* ≤ 0.007), CIS 72.76% (*p* ≤ 0.02), or CARB 72.30, (*p* ≤ 0.002). In the KLF9-overexpressing condition, RPMI-7951 cells exhibited significantly lower viability than naïve and ctrl-ad cells, where TMZ, CIS, or CARB reduced cell viability to 47.70% (*p* ≤ 0.001), 48.32% (*p* ≤ 0.001), or 48.63% (*p* ≤ 0.001), respectively. In naïve A375 cells, TMZ, CIS, or CARB reduced cell viability to 79.16% (*p* ≤ 0.02), 78.79% (*p* ≤ 0.03), or 80.80% (*p* ≤ 0.006), respectively. In ctrl-ad A375 cells, TMZ, CIS, or CARB reduced cell viability to 78.77% (*p* ≤ 0.03), 82.15% (*p* ≤ 0.01), or 82.84% (*p* ≤ 0.002), respectively. In KLF9-overexpressing A375 cells, TMZ, CIS, or CARB had greater effects compared to naïve or ctrl-ad cells and significantly reduced cell viability to 65.73% (*p* ≤ 0.007), 64.93% (*p* ≤ 0.04), or 66.58 (*p* ≤ 0.04), respectively.

Taken together, data collected from the tested malignant melanoma cell models confirmed that KLF9 overexpression potentiated the anti-proliferative effects of chemotherapeutic agents in malignant melanoma cell lines.

**Figure 7:**
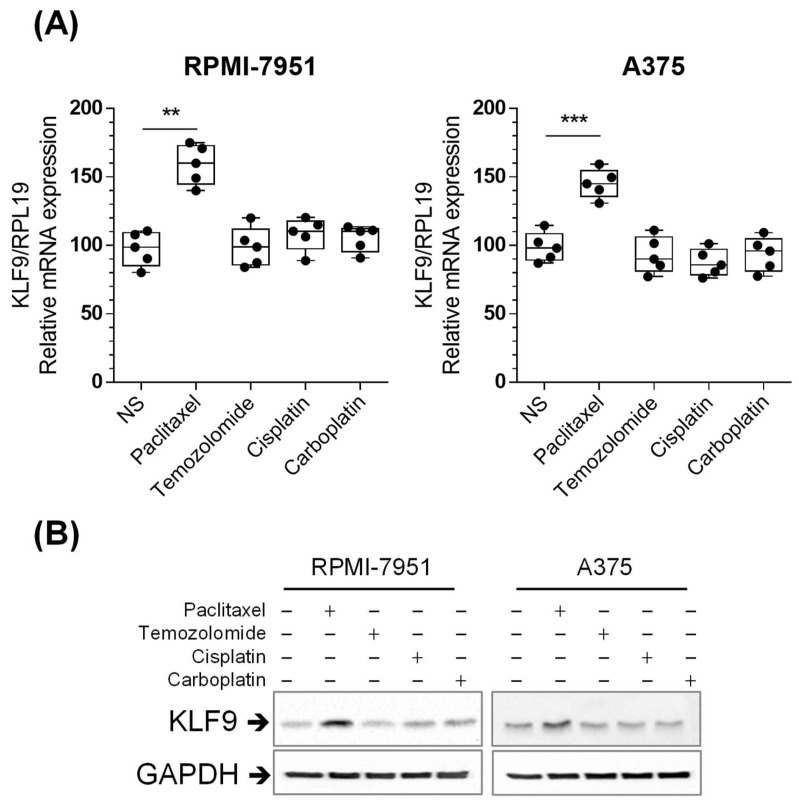
TMZ, CIS, or CARB did not change mRNA or protein levels of KLF9 in malignant melanoma-derived cell lines. RT-PCR (**A**) and Western blot analysis (**B**) illustrate the differential effects of four chemotherapeutic agents on KLF9 mRNA and protein levels in RPMI-7951 and A375 cells. Data (*n* = 5), significant difference was determined using ANOVA with Tukey’s correction for multiple comparisons. ** *p* ≤ 0.01, *** *p* ≤ 0.001 vs. non-stimulated cells.

We also tested the effect of TMZ, CIS, or CARB on mRNA and protein expression of KLF9 in RPMI-7951 and A375. The data revealed that, unlike paclitaxel, there was no significant effect of TMZ, CIS, or CARB on KLF9 mRNA or protein levels (Figure 7).

## 3. Discussion

Our data confirm that paclitaxel selectively reduced the cell viability of malignant melanoma cell lines, RPMI-7951 and A375, but not primary melanocytes. These results may be attributed to the higher mitotic division rate in malignant cells, where paclitaxel induces mitotic arrest via the activation of spindle assembly checkpoints in the dividing cells [19,24]. We also observed that A375 cells are more resistant to paclitaxel than RPMI-7951, which is consistent with a previous study that compared A375 cell tolerance to paclitaxel with other malignant melanoma cells [25].

Cancer cells have developed a way to maintain a balance between high levels of reactive oxygen species (ROS) produced from their fast metabolic rates and the effect of ROS on increasing antioxidant gene expression. This is achieved by decreasing the expression of KLF9, which regulates several antioxidant agents in a negative manner [12]. Downregulation of KLF9 was also confirmed when we compared the levels of KLF9 protein in primary melanocytes versus malignant melanoma cell lines and observed the significant reduction in RPMI-7951 and A375 cells (Figure S2). Herein, we demonstrated that paclitaxel treatment increased the mRNA and protein levels of KLF9 in malignant melanoma cell lines. These results may add another mechanistic explanation to paclitaxel’s antitumor properties via promoting oxidative stress in malignant melanoma cells where KLF9 levels are proven to be downregulated [12,26].

In the literature, the role of KLF9 in melanoma progression is controversial. Some studies suggest that KLF9 does not interfere with primary tumor growth and its deficiency inhibits premalignant melanocytic hyperplasia in mice. However, other studies highlighted the importance of KLF9-dependent upregulation of ROS in controlling tumor progression via oxidative stress [12,27,28]. Thus, we investigated the role of KLF9 in paclitaxel treatment in two melanoma-derived cell lines systems, where KLF9 is knocked down in one and overexpressed in the other. According to our data, overexpression of KLF9 significantly enhanced the effects of paclitaxel. We observed a decrease in cell viability, activation of pro-caspase-3, an increase in the number of Annexin V positive cells, and a reduction in the nuclear proliferation marker (KI67). On the other hand, when KLF9 was knocked down, we observed attenuation in the effects of paclitaxel in RPMI-7951 and A375 cells. Resistance or poor treatment outcomes to paclitaxel were previously reported in A375 cells [25]. In that study, paclitaxel and retinoic acid combined treatment increased the anticancer effects of paclitaxel. Interestingly, in another study, KLF9 upregulated the expression of the retinoic acid metabolizing enzyme (cytochrome P450 26A1) [29], which is involved in all-trans retinoic acid, a signaling molecule that binds to retinoic acid receptors and regulates gene transcription. In another study, all-trans retinoic acid has been used to treat acute promyelocytic leukemia [30]. Together, these studies and our data suggest that KLF9 plays a role in sensitizing transformed cells to paclitaxel treatment and ultimately in controlling malignancy.

We further studied the effects of temozolomide, cisplatin, and carboplatin on KLF9 mRNA and protein expression, and on cell viability in RPMI-7951 and A375 cells. The results demonstrated that paclitaxel had the most potent effect on reducing cell viability in both cell types. Similar results were also reported in human malignant glioblastoma and rat glioma cells, where paclitaxel had a more substantial growth inhibitory effect than temozolomide [31]. Other studies demonstrated that paclitaxel had a higher disease control rate than temozolomide or carboplatin in melanoma patients [32,33,34]. Surprisingly, KLF9 mRNA levels did not change after treatment with temozolomide, cisplatin, or carboplatin. These results suggest that the more potent effect of paclitaxel, compared to the other three agents, may be attributed to paclitaxel-induced upregulation of KLF9. Therefore, we hypothesized that the anti-proliferative effects could be potentiated if the levels of KLF9 increased. As expected, cell viability significantly decreased in KLF9 overexpressing RPMI-7951 and A375 cells treated with temozolomide, cisplatin, or carboplatin compared to naïve, or cells that received control-adenovirus.

Numerous studies highlight the potential therapeutic benefits of paclitaxel, temozolomide, cisplatin, and carboplatin in treating malignant melanoma [33,34,35]. Our study is the first to show that KLF9 might be a significant factor in modulating the antitumor properties of these chemotherapeutic agents. With the success of COVID-19 vaccinations based on both the adenovirus vector as well as lipid nanoparticle encapsulated mRNA, it is possible to directly translate strategies in this paper into the clinical setting. For instance, a combination treatment with both paclitaxel as well as KLF9 overexpression could represent an effective treatment option for melanoma patients who are not candidates for immunotherapy or other targeted therapies. Furthermore, such a treatment strategy may represent a novel treatment option for other paclitaxel-responsive cancers.

The “hallmarks” of cancer are characterized by the ability to sustain proliferative signaling, evade growth suppressors, avoid immune destruction, enable replicative immortality, promote tumor inflammation, activate invasion and metastasis, induce angiogenesis, genomic instability and mutations, resist cell death, and deregulate cellular energetics [36]. Mechanism-based targeted therapies have revolutionized the treatment of many cancers. However, cancers can take advantage of multiple “hallmarks”, suggesting that a multi-pronged approach may increase efficacy as well as durability of response [36]. For instance, dabrafenib and trametinib target proliferative signaling, while nivolumab and ipilimumab impact immune destruction pathways. We propose that KLF9-directed therapy may serve as a therapeutic option that targets the “hallmark” of resisting cell death.

## 4. Materials and Methods

### 4.1. Cell Culture and Reagents

Primary human melanocytes (catalog no. psc-200-013; ATCC, Manassas, VA, USA) were cultured in Cascade Biologics Epilife^®^ basal medium, supplemented with melanocyte growth kit (catalog no. psc-200-041, ATCC). Human malignant melanoma cell lines, RPMI-7951 (catalog no. htb-66; ATCC), and A375 cells (catalog no. crl-1619; ATCC) were cultured in DMEM medium (catalog no. 12-604f; Lonza, Basel, Switzerland), supplemented with 15% fetal bovine serum (catalog no. 098105; Multicel, St-Bruno, QC, Canada). Paclitaxel (catalog no. T7402; Millipore Sigma, Burlington, MA, USA) was dissolved in dimethyl sulfoxide (DMSO, catalog no. d5879; Sigma-Aldrich, St. Louis, MO, USA) at a concentration of 50 mg/mL. Temozolomide (catalog no. T2577; Sigma-Aldrich) was dissolved in DMSO at a concentration of 10 mg/mL, Cisplatin (catalog no. PHR1624; Supelco, Oakville, ON, Canada) was dissolved in DMSO at a concentration of 100 mg/mL, and Carboplatin (catalog no. C2538; Sigma-Aldrich) was dissolved in water at a concentration of 10 mg/mL.

### 4.2. KLF9 Adenovirus Transduction and mRNA Transfection

To establish an adenoviral system that resulted in the overexpression of KLF9 protein in mammalian cells, KLF9 cDNA was cloned downstream to the CMV promotor in the adenoviral genome using a modified version of the AdEasy protocol (Luo et al., 2007). Briefly, KLF9 cDNA was liberated from a commercial vector (Origene, Rockville, MD, USA) using KpnI (catalog no. R3142M) and XhoI (catalog no. R0146M) enzymes (both from New England Biolabs, Whitby, ON, Canada). The pShuttle-CMV (catalog no. 50-125-389; Qbiogene, Carlsbad, CA, USA) vector was digested using the same enzymes, and both fragments were ligated using T4 DNA ligase (catalog no. M0202M; New England Biolabs) to generate pShuttle-CMV-KLF9. The ability of this vector to overexpress KLF9 was confirmed by transient transfection in RPMI-7951 and A37 cells followed by Western blot analysis of KLF9. The pShuttle-CMV-KLF9 vector was then linearized, using PmeI (catalog no. R0560S; New England Biolabs), and phosphatase-treated (using Antarctic Phosphatase, catalog no. M0289S; New England Biolabs) before being co-transformed with pAdEasy (catalog no. 50-125-387; Qbiogene) vector into BJ5183 competent cells (catalog no. NC0585359; Agilent, Santa Clara, CA, USA), allowing for homologous recombination of the two vectors. Recombinant pAd-KLF9 vector was then linearized using PacI (catalog no. R0547S; New England Biolabs) and transfected in HEK293A (catalog no. R70507; Thermo, Markham, ON, Canada) cells using Lipofectamine 2000 (catalog no. 11668019; Thermo). HEK293A cells allow the formation and propagation of packaged viral particles via acting as a donor of the early genes, E1 and E3, which were deleted in the pAdEasy vector. HEK293A cells transfected with pAd-KLF9 were incubated for 10–14 days until the appearance of the cytopathic effect (CPE) due to production and release of viral particles. Packaged adenoviral particles (Ad-KLF9) were released from cells by performing 4 freeze–thaw–vortex cycles and the supernatant, containing the viral particles, was collected by centrifugation. HEK293A cells were then infected with serial dilutions (10^−3^–10^−7^) of the supernatant before cells were layered with 1.25% SeaPlaque agarose (catalog no. 50101; Lonza) in DMEM growth medium (Catalog no. 12-604f; Lonza). Cells were incubated for 14–21 days until isolated plaques that were 2–4 mm in diameter were observable in at least one dilution. HEK293A cells were then infected with different clones of adenoviral particles, each produced from a single plaque (single infection event). Upon the appearance of CPE in infected HEK293A cells, viral particles were released by freeze–thaw–vortex cycles and Western blot analysis for KLF9 protein was performed in cell pellets. The supernatant from a clone that demonstrated successful KLF9 overexpression in the cell pellet, was used for large scale amplification of the virus in HEK293A cells. Viral particles produced from infecting 3 × 10^8^ HEK293A cells were pooled and purified using the double cesium chloride (CsCl) gradient method. Cesium chloride was then removed by dialysis. The titer (plaque forming unit; PFU) of the dialyzed virus was determined by performing a plaque assay in HEK293A cells.

### 4.3. Cell Viability Assay

Thiazolyl blue tetrazolium bromide (MTT, catalog no. m-5655; Sigma) was dissolved in phosphate buffered saline at a concentration of 5 mg/mL. After treatment, MTT was added to each well of 48-well plates at a final concentration of 1 mg/mL. Plates were then incubated at 37 °C for three hours, the culture medium was removed and MTT formazan crystals were dissolved in 300 µL MTT solvent (4 mM HCL, catalog no. acs393; BDH, 0.1% Nonidet p-40, catalog no. 74385; Fluka, in isopropyl alcohol, catalog no. un1219; Omnisolv). Plates were read on a SPECTRAmax PLUS384 Microplate spectrophotometer set to 590 nm wavelength.

### 4.4. RNA Purification, cDNA Synthesis, and QRT-PCR Analysis

Primary melanocytes, RPMI-7951 and A375 cell lines were grown in 24-well plates. After treatment, total RNA was purified using NucleoSpin RNA purification kits (catalog no. 740955-250; D-MARK Biosciences, Düren, Germany) following the manufacturer’s instructions. For cDNA synthesis, we used 1 µg of total RNA and a qScript cDNA Synthesis kit (catalog no. CA101414-098; Quanta Biosciences, Beverly, MA, USA). RT-PCR was performed using Fast SYBR Green master mix (catalog no. 4385618; Life Technologies, Carlsbad, CA, USA) and a StepOne Plus thermal cycler (Applied Biosystems, Foster City, CA, USA). PCR program parameters were 95 °C for 20 s followed by forty cycles of 95 °C for 3 s and 60 °C for 30 s. Primer sequences used were KLF9 (forward 5′ CCT CCC ATC TCA AAG CCC ATT 3′, reverse 5′ TCG TCT GAG CGG GAG AAC TT 3′), RPL19 (forward 5′ ATC GAT CGC CAC ATG TAT CA 3′, reverse 5′ GCG TGC TTC CTT GGT CTT AG 3′).

### 4.5. Cell Lysate Preparation, Total Protein Quantification, and Western Blot Analysis

After treatment, cells were harvested in RIPA buffer containing 1× protease inhibitor cocktail (catalog no. PI-78439c; Thermo Scientific, Waltham, MA, USA). Total protein concentration was determined using the BioRad protein assay (cat no. 500-0006; BioRad, Hercules, CA, USA) following the manufacturer’s protocol. Then 50 µg of total protein was loaded into SDS-PAGE and separated protein bands were transferred to nitrocellulose membrane (catalog no. rpn203D; EG Healthcare, Staten Island, NY, USA). Membranes were immunoprobed with anti-KLF9 (catalog no. sc376422; Santacruz, Heidelberg, Germany), rabbit polyclonal anti caspase-3 (catalog no. aap-113; Stressgen, Victoria, BC, Canada) and anti-glyceraldehyde-3-phosphate dehydrogenase (GAPDH; catalog no. 4699-9555; Biogenesis, Paterson, NJ, USA). To detect the immuno-probed protein bands we used the secondary antibody peroxidase-affiniPure goat anti-mouse IgG (catalog no. 115-035-003; Jackson ImmunoResearch, Ely, UK). Band visualization and densitometric analysis were carried out using Pierce ECL Western Blotting Substrate (catalog no. PI-32106; Thermo Scientific), ChemiDoc XRS system and image Lab 6.0 software (BioRad).

### 4.6. Annexin V Staining and Flow Cytometry Analysis

To identify apoptotic cells, Annexin V staining (Catalog no. A13199; ThermoFisher) was performed according to the manufacturer’s instructions. Stained cells were analyzed using flow cytometry (Guava^®^ easyCyte; MilliporeSigma, Burlington, MA, USA). Data analysis was carried out using WinMDI software.

### 4.7. Immunofluorescent Microscopy and Image Analysis

Immunofluorescent staining was performed following previously published protocols [37]. Briefly, cells were grown on coverslips in 12-well tissue culture plates. After treatment, the cell monolayers were fixed in ice-cold methanol (catalog no. A412; Fisher Chemicals, Waltham, MA, USA) for 10 min at −20 °C. To minimize background fluorescence, cells were incubated in 1% BSA (catalog no. a-4503; Sigma-Aldrich) dissolved in PBS containing 0.01% Tween 20 (catalog no. P5927; Sigma-Aldrich) for 1 h. Immunostaining was carried out by incubation of the cell monolayers with the manufacturer recommended concentrations of antibodies against KLF9 (mouse monoclonal, catalog no. sc376422; Santacruz), and rabbit KI67 antibody (catalog no. ab15580; Abcam) prepared in PBS-Tween-BSA buffer. For the detection of the interactive primary antibodies, we used Alexa Fluor 488 goat anti-mouse (catalog no. A11029; Invitrogen, Burlington, ON, Canada), and Alexa Fluor 568 goat anti-rabbit IgG (H + L) (catalog no. a11011; Invitrogen) secondary antibodies. For a nuclear marker, we used 4′,6-diamidino-2-phenylindole (catalog no. d21490; Molecular Probes) at a concentration of 300 nM in PBS and incubated the cell monolayers for 5 min at room temperature. Fluorescence visualization was carried out using an OLYMPUS IX73 inverted fluorescent microscope and cellSens Dimension 1.9 software. KI67 fluorescence quantification were carried out using imagej online software “http://imagejs.org/ (accessed on15 November 2021)”.

## 5. Conclusions

We propose that restoring levels of KLF9 may sensitize melanoma cells to the chemotherapeutic drug, paclitaxel. Experiments using adenovirus overexpression and siRNA technologies were conducted to assess the role of KLF9 in melanoma cells’ response to paclitaxel treatment. The results suggest that increasing KLF9 levels enhances the effectiveness of paclitaxel, as evidenced by increased cell death and decreased proliferation markers in cells overexpressing KLF9. These findings suggest that KLF9 serves as a potential target for improving patient chemotherapeutic response in malignant melanoma.

## Data Availability

Data are contained within the article and the Supplementary Materials.

## Supplementary Materials

The following supporting information can be downloaded at: https://www.mdpi.com/article/10.3390/ph16040557/s1, Figure S1: Differences in growth rate in primary melanocytes and malignant melanoma-derived cell lines; Figure S2: Downregulation of KLF9 in malignant melanoma derived cell lines compared to primary melanocytes; Table S1: IC50 data analyzed; Table S2: Quantification of Ki67 positive nuclei in (RPMI-7951); Table S3: Quantification of Ki67 positive nuclei in (A375 cells).
