# Supplementary material for: Overexpression of Krüppel-Like Factor 9 Enhances the Antitumor Properties of Paclitaxel in Malignant Melanoma-Derived Cell Lines
Source: Pharmaceuticals (Basel). 2023 Apr 6;16(4):557. doi: 10.3390/ph16040557 (PMC10144003; doi:10.3390/ph16040557)
Supplement: Supplementary file 1 [file pharmaceuticals-16-00557-s001.zip › pharmaceuticals-2244989-supplementary.pdf]

pharmaceuticals

MDPI

Supplementary Materials

Overexpression of Krüppel-like Factor 9 Enhances the Antitumor Properties of Paclitaxel in Malignant Melanoma-Derived Cell Lines

Mohammed O. Altonsy [1,2,3,*], George X. Song-Zhao [1], Mahmoud M. Mostafa [2] and Paule Régine Mydlarski [1,2,*]

[1] Division of Dermatology, Department of Medicine, University of Calgary, Calgary, AB T2T 5C7, Canada; george.songzhao@ucalgary.ca

[2] Snyder Institute for Chronic Diseases, University of Calgary, Calgary, AB T2N 4N1, Canada; mahmoud.mostafa@ucalgary.ca

[3] Department of Zoology, Faculty of Science, Sohag University, Sohag 82524, Egypt

* Correspondence: mohammedomarahmed.mo@ucalgary.ca (M.O.A.); regine.mydlarski@ucalgary.ca (P.R.M.); Tel.: +1-403-220-6848 (M.O.A.); +1-403-955-8345 (P.R.M.); Fax: +1-403-955-8200 (P.R.M.)

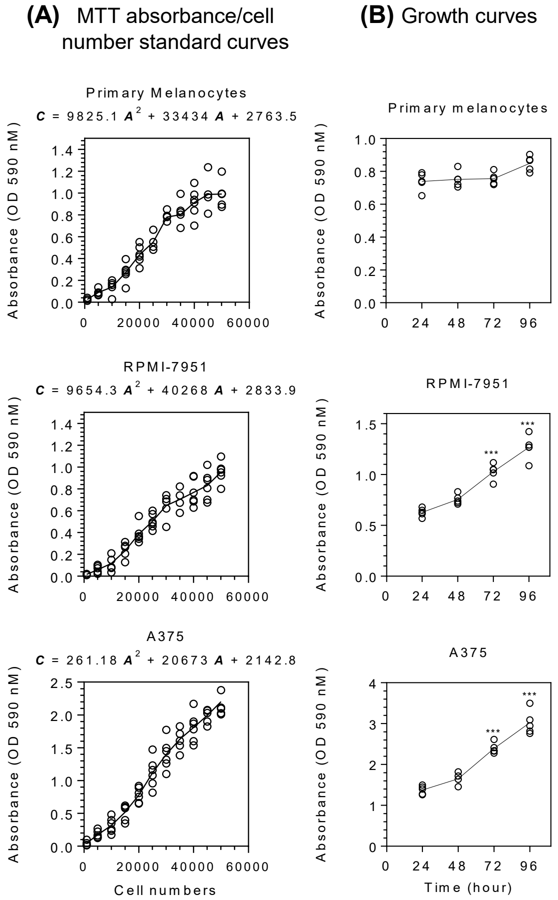

Figure S1. Differences in growth rate in primary melanocytes and malignant melanoma-derived cell lines. (A) MTT absorbance values, [*A*], versus cell counts, [*C*] standard curves. Cells were plated at varied numbers, and MTT absorbance values were measured and plotted to extract a second-degree polynomial function interpolating cell numbers for a given absorbance value by using the standard curve equation. **(B)** MTT assay demonstrating the differences in growth rate among primary melanocytes, RPMI-7951 and A375 cell types. Data were collected at multiple time points over four consecutive days. Absorbance was measured at 590 nM, Data (n = 5-6) and expressed as scatter plots or or mean OD values with SD, using Graph-Pad Prism software. ***$P \leq 0.001$ *vs*. the absorbance values measured at the first time point.

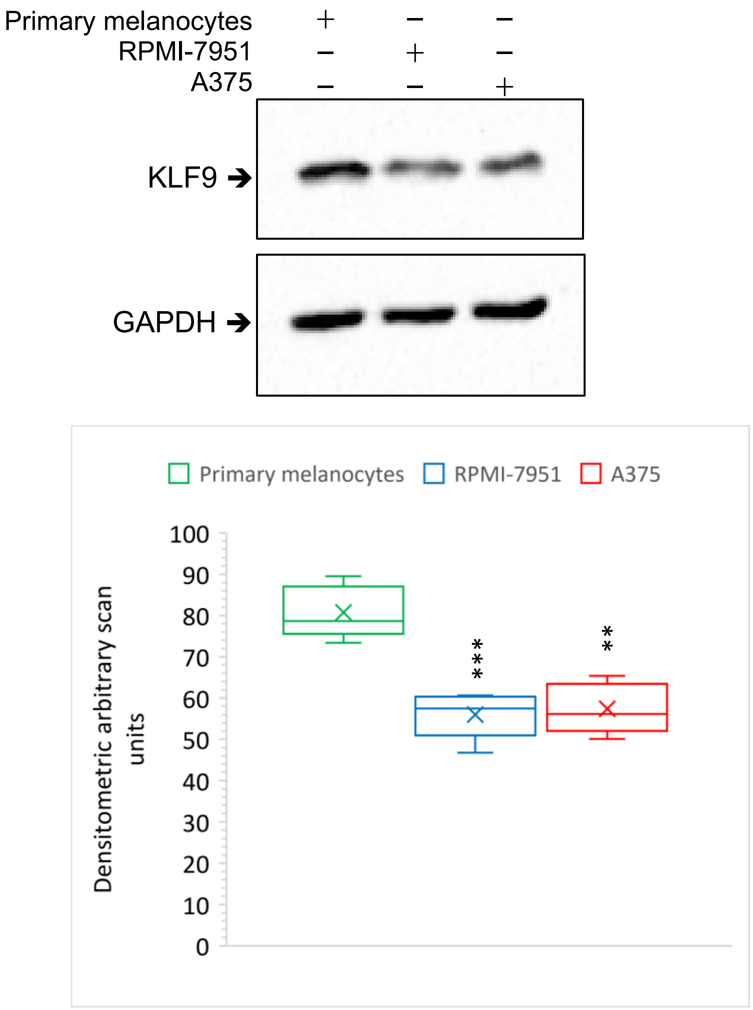

Figure S2. Downregulation of KLF9 in malignant melanoma derived cell lines compared to primary melanocytes. Western blot (top) and densitometric analysis (bottom panel) of protein lysates extracted from primary melanocytes, RPMI-7951 and A375 cells. Total protein, 50 µg, were loaded to SDS PAGE. Separated protein bands were transferred to nitrocellulose membranes and immune-stained against KLF9 or GAPDH antibodies. Protein band visualization was carried out using image Lab 6.0 software and ChemiDoc XRS system (BioRad). KLF9 Band intensities were normalized to GAPDH of the same sample and depicted as arbitrary scan units, box-and-whiskers plot. Significance was determined using repeated-measure, one way analysis of variance (ANOVA) with Tukey's correction for multiple comparison. Data (n = 5), ** $P \leq 0.01$, *** $P \leq 0.001$ *vs*. the densitometric values of KLF9 protein bands in primary melanocytes.

Table S1. IC_{50} data analyzed: Paclitaxel IC_{50} Calculations using Graph-Pad Prism software (Restricted cubic spline curve generated. 36 points were calculated with the X values (paclitaxel cincentration) ranging from 0 to 5000.

24 h	A375		RPMI-7951	
**Reblicates**	**IC_{50} (nM)**	**SEM**	**IC_{50} (nM)**	**SEM**
1	171.638		171.196	
2	108.290		140.502	
3	146.274	10.40	168.692	5.91
4	130.639		147.602	
5	145.560		157.097	
**Mean**	**140.481**		**157.018**	

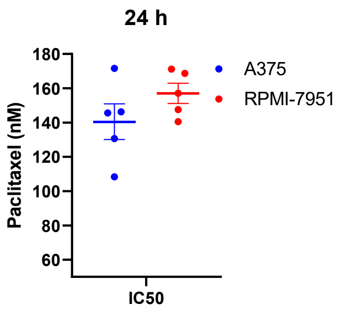

48 h	A375		RPMI-7951	
**Reblicates**	**IC_{50} (nM)**	**SEM**	**IC_{50} (nM)**	**SEM**
1	105.022		49.028	
2	96.680		61.023	
3	96.1638	3.977	59.271	2.218
4	115.511		55.2827	
5	112.660		52.057	
**Mean**	**105.208**		**55.33**	

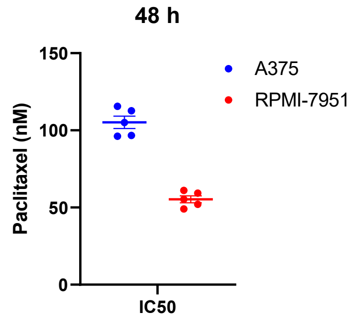

72 h	A375		RPMI-7951	
**Reblicates**	**IC_{50} (nM)**	**SEM**	**IC_{50} (nM)**	**SEM**
1	78.018		28.948	
2	72.508		27.335	
3	69.042		35.377	
4	84.285	2.739	34.233	1.532
5	71.391		32.345	
**Mean**	**75.049**		**31.648**	

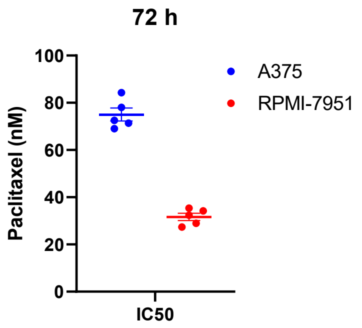

Table S2. Quantification of Ki67 positive nuclei in (RPMI-7951).

	(-) Paclitaxel				(+) Paclitaxel			
	**Representative image analysis**	**All nuclei**	**Ki67 positive nuclei**	**Ki67 positivity %**	**Representative image analysis**	**All nuclei**	**Ki67 positive nuclei**	**Ki67 positivity %**
**Naive**	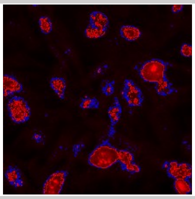	15.26	7.61	49.86	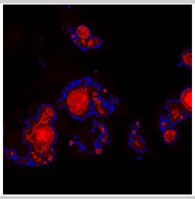	14.26	4.55	31.90
		17.13	9.67	56.45		15.69	6.42	40.91
		15.54	6.43	41.37		16.02	5.46	34.08
		16.56	8.55	51.63		13.11	4.56	34.78
		14.88	7.79	52.35		14.17	4.19	29.56
		18.12	7.44	41.05		12.55	3.8	30.27
	Mean	***16.24***	***7.91***	***48.78***	*Mean*	***14.3***	***4.83***	***33.58***
**Ctrl-Ad**	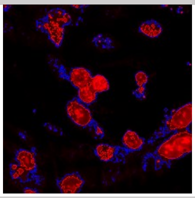	18.15	9.79	53.93	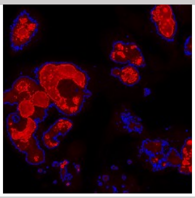	25.65	8.01	31.22
		15.78	8.37	53.04		20.64	6.63	32.12
		15.69	8.86	56.46		19.24	7.47	38.82
		14.09	8.41	59.68		19.15	5.16	26.94
		17.6	8.09	45.96		14.13	5.01	35.45
		15.61	7.67	49.13		15.93	4.42	27.74
	Mean	***16.15***	***8.53***	***53.03***	*Mean*	***19.12***	***6.11***	***32.05***
**KLF9-Ad**	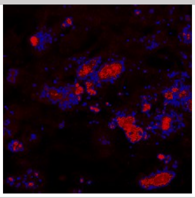	14.63	5.86	40.05	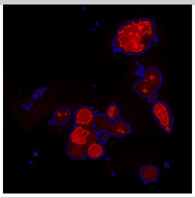	13.69	2.61	19.06
		11.26	4.25	37.74		20.09	4.34	21.60
		12.41	6.3	50.76		11.17	2.03	18.17
		15.72	6.33	40.26		20.58	4.21	20.45
		13.89	5.53	39.81		11.91	2.07	17.38
		14.75	5.01	33.96		13.05	3.71	28.42
	Mean	***13.77***	***5.54***	***40.43***		***15.08***	***3.16***	***20.85***
**Ctrl-siRNA**	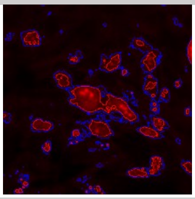	17.63	8.22	46.62	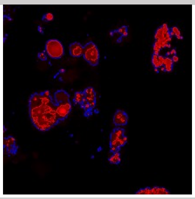	11.94	4.83	40.45
		16.01	8.22	51.34		12.22	5.15	42.14
		18.52	8.87	47.89		14.28	5.07	35.50
		20.11	9.85	48.98		15.8	5.95	37.65
		15.63	7.85	50.22		14.87	5.9	39.67
		17.85	10.12	56.69		14.08	4.29	30.46
	Mean	***17.62***	***8.85***	***50.29***	*Mean*	***13.86***	***5.19***	***37.65***
**KLF9-siRNA**	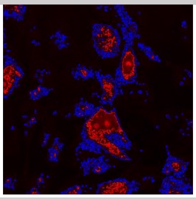	16.92	10.47	61.87	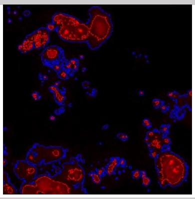	13.67	6.57	48.06
		20.05	11.93	59.50		13.89	7.06	50.82
		18.05	11.19	61.99		15.93	8.13	51.03
		17.45	11.17	64.01		13.93	7.62	54.70
		17.16	9.82	57.22		11.79	5.53	46.90
		16.11	10.71	66.48		11.59	5.42	46.76
	Mean	***17.62***	***10.88***	***61.84***	*Mean*	***13.46***	***6.72***	***49.71***

Table S3: Quantification of Ki67 positive nuclei in (A375 cells)

	(-) Paclitaxel				(+) Paclitaxel			
	**Representative image analysis**	**All nuclei**	**Ki67 positive nuclei**	**Ki67 positivity %**	**Representative image analysis**	**All nuclei**	**Ki67 positive nuclei**	**Ki67 positivity %**
**Naive**	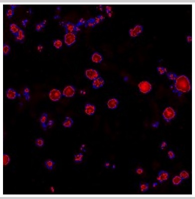	7.99	4.75	59.44	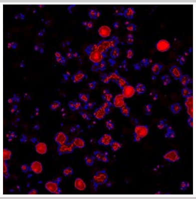	15.47	6.7	43.30
		11.48	5.63	49.04		16.42	7.81	47.56
		13.6	7.41	54.48		14.67	5.95	40.55
		17.12	8.91	52.04		15.64	5.96	38.10
		16.83	10.36	61.55		19.88	9.07	45.62
		19.07	12.84	67.33		19.79	9.17	46.33
	Mean	***14.34***	***8.31***	***57.31***	*Mean*	***16.97***	***7.44***	***43.58***
**Ctrl-Ad**	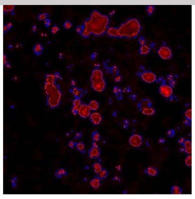	13.87	7.46	53.78	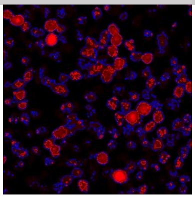	25.73	8.24	32.02
		17.58	9.03	51.36		20.73	8.54	41.19
		14.7	8.41	57.21		18.94	6.92	36.53
		15.27	7.68	50.29		17.17	6.57	38.26
		11.38	6.19	54.39		15.25	6.58	43.14
		12.91	7.82	60.57		16.08	7.19	44.71
	Mean	***14.28***	***7.76***	***54.60***	*Mean*	***18.98***	***7.34***	***39.31***
**KLF9-Ad**	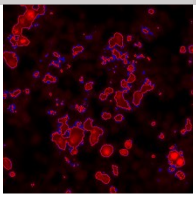	4.07	1.67	41.03	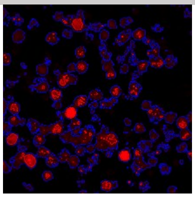	6.17	1.71	27.71
		10.14	4.43	43.68		10.69	3.98	37.23
		12.02	5.11	42.51		7.76	2.43	31.31
		9.7	5.04	51.95		7.71	2.38	30.86
		11.63	5.37	46.17		11.91	3.18	26.70
		14.33	5.73	39.98		15.42	4.17	27.04
	Mean	***10.31***	***4.55***	***44.22***		***9.94***	***2.97***	***30.14***
**Ctrl-siRNA**	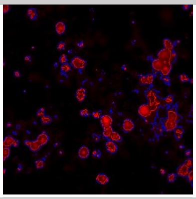	13.45	6.61	49.14	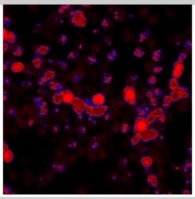	15.02	6.86	45.67
		12.93	7.32	56.61		11.72	5.01	42.74
		9.06	4.68	51.65		14.08	5.8	41.19
		11.25	5.49	48.80		10.9	5.04	46.23
		15.34	6.79	44.26		9.02	3.51	38.91
		13.03	6.94	53.26		16.27	7.08	43.51
	Mean	***12.51***	***6.30***	***50.62***	*Mean*	***12.83***	***5.55***	***43.04***
**KLF9-siRNA**	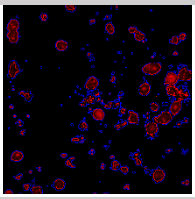	14.68	10.23	69.68	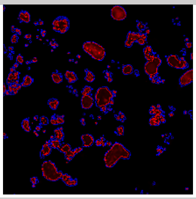	11.12	7.57	68.07
		10.5	7.04	67.04		10.82	6.54	60.44
		15.07	10.03	66.55		10.24	6.03	58.88
		12.09	7.77	64.26		8.48	3.93	46.34
		14.7	7.35	50.01		12.09	7.27	60.13
		13.2	7.42	56.21		14.28	7.52	52.66
	Mean	***13.37***	***8.30***	***62.29***	*Mean*	***11.17***	***6.47***	***57.75***
